# Effective Gene Therapy in a Mouse Model of Prion Diseases

**DOI:** 10.1371/journal.pone.0002773

**Published:** 2008-07-23

**Authors:** Karine Toupet, Valérie Compan, Carole Crozet, Chantal Mourton-Gilles, Nadine Mestre-Francés, Françoise Ibos, Pierre Corbeau, Jean-Michel Verdier, Véronique Perrier

**Affiliations:** 1 Univ Montpellier 2, Montpellier, France; 2 Inserm, U710, Montpellier, France; 3 EPHE, Paris, France; 4 Institut de Génomique Fonctionnelle, CNRS UMR 5203, 34094, Montpellier, France; 5 Institut de Génétique Humaine, CNRS UPR 1142, 34094, Montpellier, France; 6 CNRS FRE3009 BioRad, Cap Delta, 34790, Grabels, France; 7 Laboratoire de lentivirus et transfert de gènes, CNRS UPR 1142, 34396, Montpellier, France; University of Florida, United States of America

## Abstract

Classical drug therapies against prion diseases have encountered serious difficulties. It has become urgent to develop radically different therapeutic strategies. Previously, we showed that VSV-G pseudotyped FIV derived vectors carrying dominant negative mutants of the PrP gene are efficient to inhibit prion replication in chronically prion-infected cells. Besides, they can transduce neurons and cells of the lymphoreticular system, highlighting their potential use in gene therapy approaches. Here, we used lentiviral gene transfer to deliver PrPQ167R virions possessing anti-prion properties to analyse their efficiency *in vivo*. Since treatment for prion diseases is initiated belatedly in human patients, we focused on the development of a curative therapeutic protocol targeting the late stage of the disease, either at 35 or 105 days post-infection (d.p.i.) with prions. We observed a prolongation in the lifespan of the treated mice that prompted us to develop a system of cannula implantation into the brain of prion-infected mice. Chronic injections of PrPQ167R virions were done at 80 and 95 d.p.i. After only two injections, survival of the treated mice was extended by 30 days (20%), accompanied by substantial improvement in behaviour. This delay was correlated with: (*i*) a strong reduction of spongiosis in the ipsilateral side of the brain by comparison with the contralateral side; and (*ii*) a remarkable decrease in astrocytic gliosis in the whole brain. These results suggest that chronic injections of dominant negative lentiviral vectors into the brain, may be a promising approach for a curative treatment of prion diseases.

## Introduction

Transmissible spongiform encephalopathies (TSEs), or prion diseases, are fatal neurodegenerative disorders in humans and animals for which no effective therapy exists. The pathogenesis of prion diseases is based on the presence of PrP^Sc^, a protease-resistant isoform of the normal cellular prion protein called PrP^C^ [1], [2]. The progressive accumulation of PrP^Sc^ in the brains of affected individuals, which can occur for up to 20 years, is associated with the neurodegeneration.

During the last decade, the BSE crisis in Europe and the transmission of the BSE infectious agent to humans have placed prion diseases on centre stage [3]. The emergence of acquired forms of human TSEs in younger people, such as variant CJD (vCJD) and iatrogenic CJD (iCJD) resulting from contaminated cadaveric growth hormone mostly in France [4] or dura grafts in Japan [4], have highlighted the urgent need for effective treatments. Furthermore, two recent studies have reported vCJD cases likely resulting from the transfusion of prion-contaminated blood [5], [6], raising concerns about the safety of blood products as well as the possibility of a second epidemic of vCJD.

The identification of compounds possessing anti-prion activity, such as porphyrin and phtalocyanin derivatives [7] or anti-PrP antibodies [8], has allowed a substantial improvement in the survival time of animals. However, these molecules are only maximally effective when administered at or near the time of prion inoculation, before the infectious agent can reach the central nervous system. The efficacy of these compounds at early stages of the disease suggests that they might best be used for post-exposure prophylactic or preclinical treatments [9]. Unfortunately, early treatment is impossible in human patients because the disease can only be detected after the onset of neurological symptoms, even in familial cases. Recently, intraventricular infusion of Pentosan Polysulfate (PPS) into mice at 35 days post-inoculation with prions gave a delay of 36 days in the mean incubation time, but severe adverse effects in the animals were observed [10]. Administration of such a high dose in humans is not appropriate because of the risk of urinary infections, haemorrhage, seizure, and death [10], [11]. A recent article reporting the continuous intraventricular administration of approximately 1–110 µg/kg/day of PPS over 18 days in a human patient concluded that the drug cannot be used for the treatment of human TSE [12]. In addition, quinacrine and chlorpromazine have been described as efficient inhibitors of PrP^Sc^ formation in neuroblastoma cells [13], [14]. As these medications had already been approved for the treatment of malaria and various psychoses, they were administered as a “compassionate treatment” to patients suffering from sporadic CJD or vCJD. No therapeutic effect, however, was observed following quinacrine treatment in 20 patients [10], [15], [16].

It is now clear that classical drug therapy faces serious difficulties and that the time has come to examine radically different strategies. Since natural resistance to prion diseases exists in animals and humans [17]–[20] and has been extensively studied both in vitro [21], [22] and in vivo [23]–[27], we took advantage of these dominant negative inhibition properties by pursuing an innovative therapeutic concept : Can prion diseases be treated with the prion protein itself?

In a previous article, we reported that VSV-G (vesicular stomatitis virus G glycoprotein) pseudotyped FIV (Feline Immunodeficiency Virus) lentiviral vectors carrying dominant negative mutant forms of the mouse *Prnp* gene (PrPQ167R or PrPQ218K) were able to inhibit prion replication in prion-infected cells [28]. *In situ* injections performed in healthy mice showed that the lentiviral vectors could transduce both neurons and cells of the lymphoreticular system, highlighting their potential use in gene therapy approaches for the treatment of prion diseases [28]. Since treatment for prion diseases is generally initiated very late in humans, partly because of the lack of an early diagnosis test, we focused on the development of a curative therapeutic protocol targeting the late stage of the disease, either at 35 or 105 days post-infection (d.p.i.) with prions. A prolongation in the lifespan of treated mice prompted us to develop a system of cannula implantation into the brain of prion-infected mice. Chronic injections of PrPQ167R virions were done at 80 and 95 d.p.i. After only two injections, survival of the treated mice was extended by 30 days (20%), accompanied by substantial improvement in behaviour. Brain tissue analysis of mice treated with PrPQ167R virions indicated a remarkable decrease in astrocytic gliosis throughout the brain, suggesting that the PrPQ167R variant could play a key role in preventing the activation of inflammatory processes.

## Materials and Methods

### Reagents and Antibodies

Pefabloc and proteinase K were purchased from Roche. Ketamine was obtained from Mérial (Lyon, France) and xylazine from Bayer HealthCare (Puteaux, France). Anti-PrP antibodies, SAF32, SAF60, SAF69, SAF70, and SAF84 were kindly provided by Dr. Jacques Grassi (CEA, Saclay, France) [28]. Anti-GFAP antibodies were purchased from AbCys (Paris). For immunohistology, the secondary antibody was provided in the Strept ABC Complex Kit (AbCys, Paris). Immunoblot secondary antibodies were from Jackson ImmunoResearch (West Grove, PA). All other chemicals were from Sigma (Paris).

### Animal models

C57Bl/6J mice were purchased from Charles River (Arbresle, France) and Janvier (Le Genest-St-Isle, France) breeding laboratories. The Me7 prion strain was provided by Richard Carp (New York Institute, NY, USA). Mice were intracerebrally inoculated in the right parietal portion of the brain with 20 µl of 1% brain homogenate (w/v) in PBS corresponding to 2×10^5^ LD_50_. Groups of five mice were housed in cages placed in a ventilated protective cabinet. All experiments were carried out in a biohazard security level laboratory and according to ethical committee guidelines (Comité régional d'éthique de Montpellier, project agreement n° CE-LR-0609; animal experiment authorization n° 34.213). Mice were scored positively for prion disease when three signs of neurologic dysfunction were present, and when progressive deterioration of the animal was apparent according to 16 diagnostic criteria, as previously described [29], [30]. Once clinical signs were detected, the animals were sacrificed in extremis. Their brains were taken and either immediately frozen at −80°C or fixed in AntigenFix (Diapath) for immunohistochemistry analysis.

### Nomenclature

The Q/R polymorphism at codon 171 in sheep corresponds to the Q167R mutation in mice. Virions containing the PrPQ167R gene are termed ΨPrPQ167R. The control virions containing only the GFP gene are termed ΨGFP.

### Production of lentiviral vectors carrying the dominant negative PrPQ167R mutant

**Figure 1:**
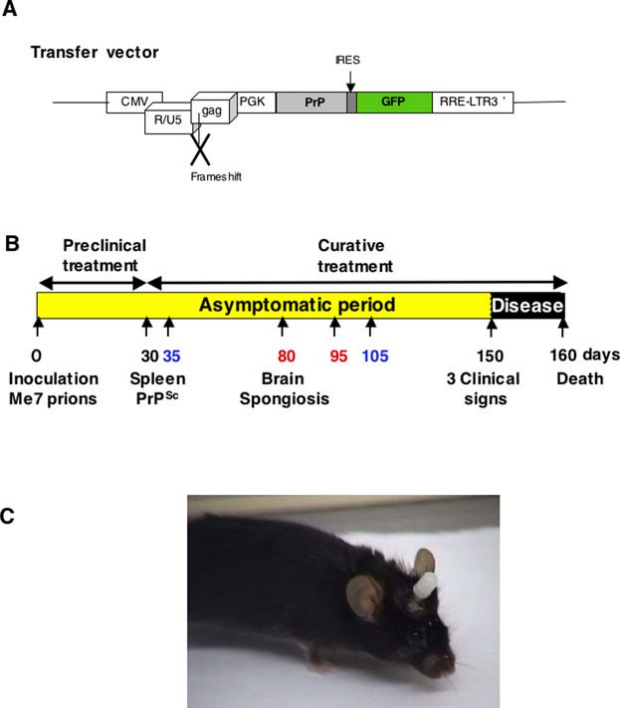
Schematic representation of the transfer vector used for virions preparation (A). The transfer vector was derived from the prototypic FIV-14-Petaluma genome (AIDS Research and Reference Reagent Program) [28]. The U3 region of the 5′ LTR was replaced with CMV promotor in order to express the vector in the cells. The *gag* gene was truncated at position 1243 and a frameshift was introduced at position 926. The *pol*, *vif*, *orfA*, *env* and *rev* genes were replaced by a cassette containing the mutated PrPQ167R gene driven by the phosphoglycerate kinase promotor (PGK). The PrP gene is followed by an IRES sequence and enhanced GFP gene. As a control vector, we used a transfer vector containing only the GFP gene without the PrP sequence. Schema of the therapeutic protocol assayed in a mouse model of prion disease (B). C57Bl/6 mice inoculated with Me7 prion strain developed a prion disease detectable after 150 days post-infection (p.i.). The prion agent first replicates in the peripheral sites, especially the lymphoreticular system, before the neuroinvasion of the central nervous system (CNS). PrP^Sc^ is detectable in the spleen at 30 days p.i. and the spongiosis is visible in the brain at about 80 days p.i. [34]. Preclinical treatments (1–30 days) target the prion agent during its replication in the lymphoreticular system and before the neuroinvasion of the CNS. Curative treatments (30–160 days) target the infectious agent after the neuroinvasion of the CNS. To evaluate the possible curative effect of ΨPrPQ167R, several protocols were tested : (*i*) a unique injection of ΨPrPQ167R at 35 or 105 days p.i. (printed in blue); and (*ii*) two administrations of the ΨPrPQ167R at 80 and 95 days p.i. (printed in red). Picture of a C57Bl/6 mouse with a guide cannula implanted into its brain (C). An obturator has been fitted into the guide-cannula to prevent the channel from being blocked by tissue regeneration.

The construction of lentiviral vectors containing the mutated gene coding for PrPQ167R and the production of lentivirus in 293T cells were performed as previously described in Crozet *et al.* (2004) [28]. The transfer vector contains the mutated PrP sequence, followed by an internal ribosome entry sequence (IRES), and the enhanced green fluorescent (GFP) protein marker gene (Figure 1A). This IRES sequence allows the determination of the infectious titer of the virions by measuring the fluorescence of the GFP proteins after transduction of 293T cells. The infectious titer of the ΨPrPQ167R, determined by FACS analysis, was 4.2×10^7^ transforming unit/ml (T.U./ml). The infectious titer of the empty virions (ΨGFP) was 9×10^9^ T.U./ml. Transduction into *Prnp^−/−^* cells (generous gift of T. Onodera) was carried out to confirm the expression of our lentivirus before performing *in vivo* experiments, as described in Crozet *et al.* (2004) [28].

### Surgery for implantation of guide cannula

Seventy days post-inoculation (p.i.) with prions, each mouse underwent surgery to implant a guide cannula into its brain (Plastics One, Roanoke,VA). Using a stereotaxic frame (Kopf Instruments, Tujunga, CA), the guide cannula was fixed in the hippocampal brain region according to the following coordinates: L: 1.2 mm; R: −1.8 mm; P: −2.0 mm (Paxinos and Franklin stereotaxic Atlas, 1997). The guide cannula allows the formation of a stable aperture in the brain through which treatments can be administered. In the absence of treatment, the guide cannula contains an obturator which prevents the aperture from being filled in by surrounding tissue. A cicatrization delay of about 10 days is necessary before realizing the first treatment.

### Administration of treatments in mice

Single injections of virions or PBS were done in the hippocampal area of mouse brain (coordinates : L: 1.2 mm; R: −1.8 mm; P: −2.0 mm Paxinos and Franklin stereotaxic Atlas, 1997) using a stereotaxic frame. At 35 or 105 days p.i. with prions, anesthetized mice received 2×10^5^ T.U. of virions or PBS, at a rate of 0.5 µl/min, allowing slow diffusion.

Groups of mice that received two injections of virions received the treatment through an internal cannula fixed to the guide and a connector linked to an automatic pump. At 80 and 95 days p.i. with prions, 2×10^5^ T.U. of ΨPrPQ167R or ΨGFP were administered *in situ* in mice brain at a rate of 0.5 µl/min, allowing slow diffusion. In addition, injections of vector doses below 1×10^8^ T.U do not induce any innate increased recruitment of inflammatory cells to the brain, and do not cause glial or neuronal toxicity [31].After surgery, each mouse was kept in an individual cage to prevent the removal of the guide cannula by other animals. Mice awoke around 30–60 min after surgery. Then, they remained under daily observation for one week for possible suffering, and twice a week until 140 days p.i. Starting at 140 days after prion infection, mice were observed daily for possible neuropathological signs. Movies of mice were made at 172 and 189 days p.i. with prions using a digital video camera recorder.

### Immunoblotting of PrP isoforms

Brain tissues were homogenized in 10% (w/v) PBS using microbead-containing tubes and a Ribolysor apparatus (Biorad, Marnes-la Coquette, France). Samples were shaken for 45 sec. and supernatants collected through an insulin syringe (Terumo) to obtain a homogeneous suspension. Proteinase K digestion of brain samples was performed as described [27]. Undigested samples were prepared by mixing an aliquot of 10% brain homogenate with an equal volume of SDS loading buffer and were then boiled for 5 min. Thirty microliters of the PK-treated or undigested samples were loaded onto 12% SDS/PAGE Precast gels (Criterion system from BioRad). Immunoblots were performed using standard procedures. The PrP^Sc^ protein was detected using a mixture of three monoclonal antibodies (SAF60, SAF69 and SAF70), called SAFmix, as previously described [32]. The normal PrP^C^ protein was detected with SAF32 monoclonal antibodies.

### Immunohistochemistry analysis

Brain tissue was first immersed in Antigenfix solution (DiaPath, France) for 24 h. Then, they are decontaminated for 30 min in a formic acid solution according to protocol described by Andréoletti et *al.*(2004) [33] and stored in 100 mM phosphate buffer at pH 7.4 with 0.02% azide. Samples were dehydrated in graded ethanol, cleared in cedar oil, and then embedded in paraffin. Frontal tissue sections were cut at six to ten micrometers using a microtome and mounted on superfrost plus slide glasses (Microm France, Francheville). Sections were dewaxed and stained with hematoxylin and eosin. Immuno-labelling using anti-glial fibrillary acidic protein (anti-GFAP) antibodies was performed according to the instructions provided with the Strept ABC Complex Kit. Immune reactions were visualized using 3-3′-diaminobenzidine (DAB) chromogen solution (Sigma, France). PrP^Sc^ immunohistochemistry was performed on dewaxed tissue sections, which were first immersed in pure formic acid for 5 min, rinsed in distilled water and incubated at 121°C for 1 h. Then, the slides were submitted to a proteinase K treatment (20 µg/ml) for 20 min at 37°C, rinsed in Tris Buffer Saline (TBS), and incubated overnight with the primary antibody SAF84 (1/500) at 4°C. After several rinses, the slides were incubated with the secondary antibody (anti-mouse IgG 1/100). Immune reactions were visualized using 3-3′-diaminobenzidine (DAB) chromogen solution (Sigma, France). Tissue sections of each group were treated identically with the same incubation time for comparative analysis.

### Software and statistical analysis

For animal experiments, we used the Kruskal and Wallis test with a probability of 0.05 defined as a significant difference, followed by Scheffé test used for comparison of each group of mice versus PBS-treated animals (Statview 5, Abacus concept, Berkeley, CA). Survival curves were done using GraphPad Software (San Diego, CA).

Mercator (Explora Nova, La Rochelle, France) was used for the counting of vacuoles and GFAP-labelled cells in histological sections. Following a significant one-way ANOVA, we used the Fisher PLSD test for multiple comparisons, with a probability of 0.05 defined as a significant difference.

## Results

### PrPQ167R virions increased the survival time of prions-inoculated mice

C57Bl/6 mice inoculated with the Me7 prion strain are an effective model of prion diseases that have been well described for their incubation time, vacuolization profiles, and immunohistology [34], [35]. These mice develop significant vacuolization (scored 3 out of 5) in the hippocampus, thalamus, hypothalamus, and anterior and posterior cerebral cortex (also called the secondary visual cortex). Since human patients also exhibit substantial spongiosis in their brain, this model is well adapted for evaluating therapeutic strategies. As previous injections performed in healthy mice showed the potential use of lentiviral vectors in gene therapy [28], our objectives was to administer dominant negative virions to evaluate their efficiency in the curative treatment of prion diseases (Figure 1B). We had previously shown that *in situ* injections are more efficient to transduce the parenchyma cells than the intracerebroventricular injections [28], so we decided to perform *in situ* injections into the hippocampal region. Indeed, this structure is the most affected by the spongiosis which can be detected as early as 84 days in the C57Bl/6J mice infected with the Me7 prion strain (Figure 1B) [34]. In addition, the hippocampal structure is involved in memory processes severely deteriorated in prion diseases.

Production of lentivirus was carried out and PrP expression was validated in Prnp^−/−^ cells. The dominant negative inhibition was confirmed in prion-infected cells, as described in Crozet *et al.* [28]. The possible curative effect of the treatment was assayed at two different stages: at 35 days p.i., which is the beginning of the neuroinvasion, and at 105 days p.i., once the brain tissues are already dammaged corresponding to a very late treatment [34]. At 35 or 105 days p.i. with prions, mice received 2×10^5^ T.U. of lentivirus containing either the GFP gene or the PrPQ167R gene. Mice treated with ΨPrPQ167R at 35 days p.i. exhibited a delay of 19 days (12%), which is a statistically significant difference from the PBS or ΨGFP treated groups, according to statistical analysis (*p*<0.001) (Table 1). Interestingly, the group of mice treated at 105 days p.i. seemed to have a delay of 9.3 days (6%). Only a single injection was necessary to obtain these results that encouraged us to pursue the dominant negative treatment strategy to enhance protection.

**Table 1:** Survival time after administration of treatment to mice.

Mouse Nbr.	Prions Inoculum	Cannula Fixation^A^	Treatment	Mean Survival Time days±s.e.m.	Delay vs PBS-treated mice^B^ days (%)	*p* value^G^
13	Me7	No	PBS	157.4±0.9	-	-
5	Me7	No	ΨGFP^C^	158.2±0.9	-	-
5	Me7	No	ΨPrPQ167R^C^	176.6±0.2	19.2 (12%)	*p*<0.001
4	Me7	No	ΨPrPQ167R^D^	166.7±0.6	9.3 (6%)	*p* = 0.1
7	Me7	Yes	ΨGFP^E^	164.3±3.8	6.9 (4%)	*p* = 0.08
7^F^	Me7	Yes	ΨPrPQ167R^E^	187.5±2.0	30.1 (20%)	*p*<0.001

A Guide cannulae were fixed in the hippocampal brain region of mice at 70 days p.i. with prions.

B Delay was calculated in comparison with the Me7 inoculated mice that received PBS.

C One unique injection of lentiviral treatment, either ΨPrPQ167R or empty virus ΨGFP, was administered in mice at 35 days p.i. with prions.

D One unique injection of ΨPrPQ167R was administered in mice at 105 days p.i. with prions.

E Two injections of lentiviral treatment, either ΨPrPQ167R or empty virus ΨGFP, were administered in mice at 80 and 95 days p.i. with prions through the cannula.

F One mouse did not received properly administration of the lentiviral treatment ΨPrPQ167R since its cannula was blocked, not taken for statistical analysis.

G p value was calculated using Scheffé test, by comparison of each group of mice versus PBS-treated animals.

Then, we implemented a system of guide cannula implantation to allow chronic injections directly into the brain (Figure 1C). According to the previous assays with unique injection, we choose to administer the treatment in mice at 80 and 95 days p.i both because: (*i*) it corresponds to the beginning of the spongiosis in mice brain [34]; (*ii*) the expression of PrPQ167R proteins after virions integration in cells is stable during two months, allowing the presence of dominant negative proteins in the mice brain up to the terminal stage of the disease; (*iii*) a limited number of injections can be done through the cannula because after 1 month the aperture is blocked by tissue.

Seventy days post-infection (p.i.) with prions, 22 mice underwent surgery to implant a guide cannula into the brain using a stereotaxic frame. Two mice died: one during the surgery, and the other the following day due to brain haemorrhage. The guide cannula can remain implanted in the skull for several months without any problems (Figure 1C).

**Figure 2:**
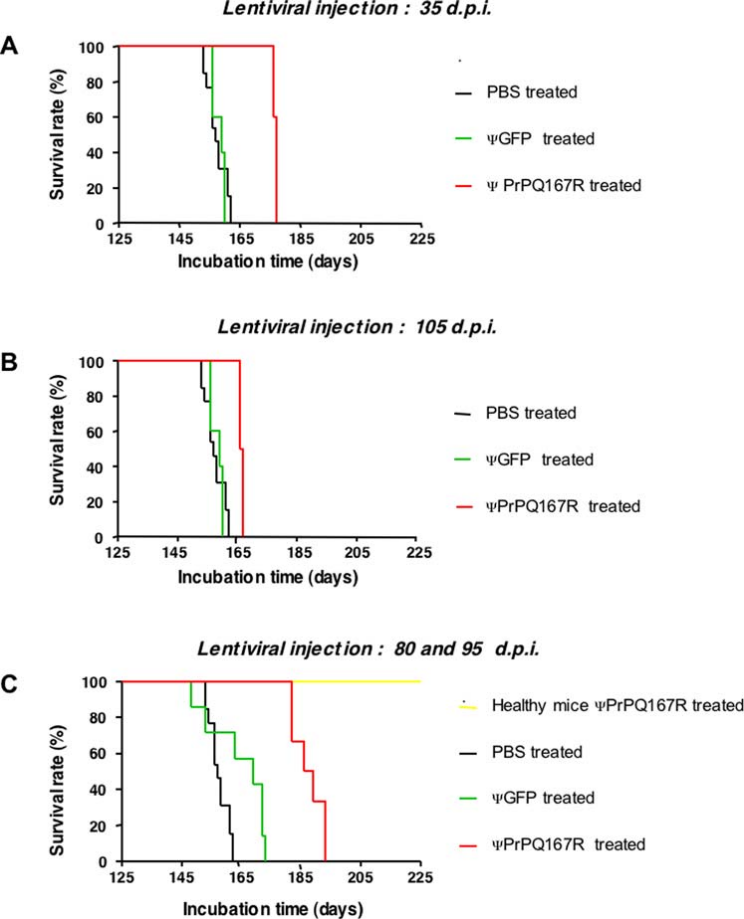
Kaplan-Meier survival plots of C57Bl/6J mice treated with lentiviral vectors. Mice inoculated with ME7 prion strain were administered with either : (A) one unique injection of PBS or ΨGFP or ΨPrPQ167R at 35 days p.i.; (B) one unique injection of PBS or ΨGFP or ΨPrPQ167R at 105 days p.i.; (C) two injections of PBS or ΨGFP or ΨPrPQ167R at 80 and 95 days p.i., through a cannula implanted in the mice brain. Healthy mice were treated with ΨPrPQ167R at 80 and 95 days p.i.

At 80 and 95 days p.i. with prions, mice received 2×10^5^ T.U. of either ΨGFP or ΨPrPQ167R (Table 1). We tried to perform a third injection but we encountered many troubles linked to the cannula completely clogged 30 days after it has been fixed on the brain. Prion-inoculated mice treated with ΨPrPQ167R showed a mean incubation time of 187.5±2.0 days. This result corresponds to a prolongation of the mean incubation time of 30.1 days (20%), which is a statistically significant difference from the PBS treated groups according to statistical analysis (*p*<0.001) (Table 1). Two mice treated with ΨPrPQ167R even survived the scrapie infection for as long as 193 days. This result was quite remarkable compared to previously reported treatment targeting the late stage of the diseases where massive dose and repeated injections or continuous administration via pump, were necessary to prolong the survival time of animals [10]. No significant differences in the mean incubation time were observed between prion-infected control mice treated with PBS (157.4±0.9 days) (Table 1) or mice implanted with a guide cannula without treatment (159.0±4.1 days, *n* = 2). This result suggests that the fixation of the guide cannula in the mouse brain did not accelerate the appearance of the clinical symptoms. Mice treated with ΨGFP showed a mean incubation time of 164.3±3.8 days (4% survival time), which was not significantly different from prion-infected control mice injected with PBS according to Scheffé test (*p* = 0.08) (Table 1). Healthy control mice received 2×10^5^ T.U. of ΨPrPQ167R at 80 and 95 days to test for possible toxicity of the treatment (*n* = 3). They did not exhibit a lethal toxic effect after treatment and were sacrificed while still healthy at 273 days after the date of prions inoculation in the other groups. Kaplan-Meier survival plots for each group illustrated the prolongation of the incubation period in all the treated animals (Figure 2 A–C).

### Improvement of behaviour in cannula-implanted mice treated with ΨPrPQ167R

Movies were successively made at 160, 172 and 189 days p.i. to compare the global behaviour of mice infected with the Me7 prion strain and administered either PBS, ΨGFP or ΨPrPQ167R, as well as healthy mouse treated with ΨPrPQ167R (see supplementary Movies S1, S2, S3 and S4). At the terminal stage of the disease, the behaviour of PBS-treated mice (movie taken at 160 days p.i.) and ΨGFP-treated mice (movie taken at 172 days which is the incubation period of the last ΨGFP-treated mouse) alternated between hyperactivity and lethargic periods during which animals were prostrated. They also exhibited kyphosis, ruffled coat, and incontinence. Mice become unbalanced and displayed combined cognitive impairments such as vision troubles and environmental perception deficits. At 160 days, mice treated with ΨPrPQ167R did not present any clinical signs. Movie taken at 172 days showed that mice treated with ΨPrPQ167R did not present the clinical signs describe above, although we did notice mild hyperactivity and visible mild ataxia when the animal was placed on top of the cage, indicating that it was already affected by the prion disease. Therefore, our results show that the dominant negative virions improved the global behaviour of the mice during the survival time.

### PrP^Sc^ deposits analysis in the brains of cannula-implanted mice treated with ΨPrPQ167R

**Figure 3:**
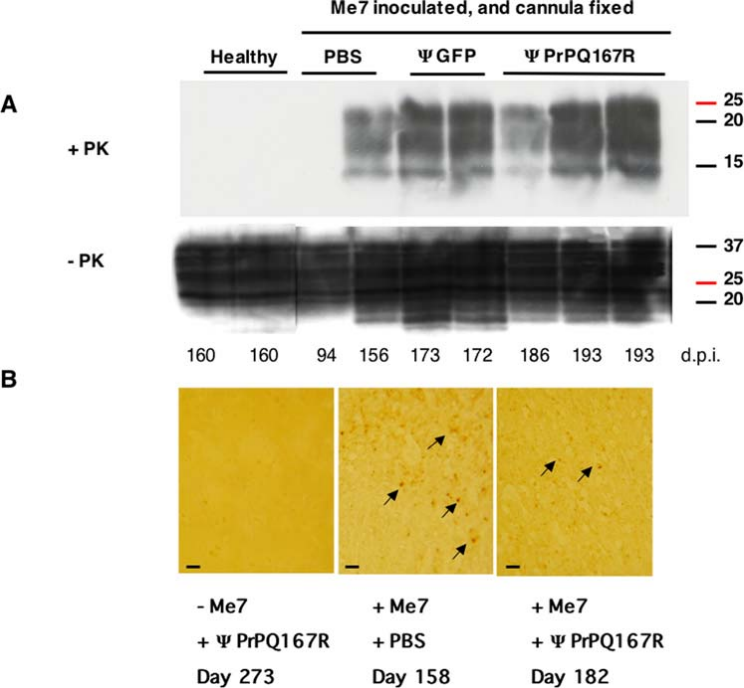
Analysis of PrP^Sc^ accumulation in the brains of mice after treatment with lentiviral vectors. Immunoblot analysis of brain homogenates before and after proteinase K digestion (A). Control mice were either not inoculated with prions or were inoculated with Me7. Mice inoculated with the Me7 prion strain and implanted with a guide cannula received injections of either PBS, lentivirus carrying the GFP gene, or lentivirus carrying the PrPQ167R gene. The numbers below indicate the dates when the mice were killed in days post-inoculation with prions (d.p.i). The numbers to the right of each blot indicate the molecular mass of the protein standards in kDa. A mix of anti-PrP antibodies was used to stain blots of SDS-polyacrylamide gels. Immunohistochemical detection of PrP^Sc^ deposits in the thalamus sections (B). The plus (+) and minus (−) signs indicate that the mice were or were not inoculated with the ME7 prion strain and whether they were injected with PBS or PrPQ167R virions. Tissue labelling was performed on 9 animals (3 per group). Pictures are representative of the staining observed in different animals in each group. Arrows indicate the localization of the PrP^Sc^ deposits in the tissue sections. Bar, 6 µm.

Brain homogenates from healthy mice treated with ΨPrPQ167R and sacrificed while they were still healthy were subjected to limited proteolysis. As expected, they showed no detectable PrP^Sc^ bands (Figure 3A). All the brain homogenates from Me7-inoculated mice treated with either PBS, ΨGFP, or ΨPrPQ167R exhibited equivalent amount of PrP^Sc^ at the terminal stage of the disease. This result is in agreement with those obtained by Genoud et al. (2008) [36], where they observed equivalent amount of PrP^Sc^ deposits at the terminal stage of the disease for ΨPrPFc-treated mice versus PBS-treated animals. Although, we noticed that one brain from a mouse treated with ΨPrPQ167R and sacrificed at 186 days showed globally lower levels of PrP^Sc^ (Figure 3A). Immunohistological analysis of brains from the first two dead mice (182 and 186 days p.i.) in the ΨPrPQ167R-treated group revealed fewer PrP^Sc^ deposits than PBS-treated mice (Figure 3B). A combination of the treatment effect with a shorter survival time in ΨPrPQ167R-treated mice (182, 186 days versus 193 days), may have limited the amount of PrP^Sc^ accumulated at the terminal stage of the disease (Figure 3A–B).

### Brain spongiosis in the vicinity of the guide cannula

**Figure 4:**
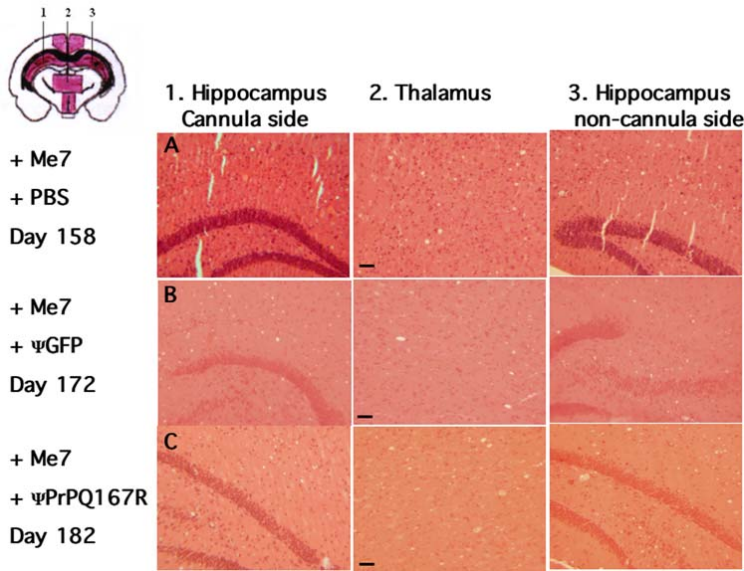
Histological analysis of brain tissue sections. All the tissue sections were stained with hematoxylin and eosin and examined histologically to confirm prion pathology as evidenced by the presence of vacuoles. The plus (+) signs indicate that the mice were inoculated with the ME7 prion strain. Mice received two injections of either PBS (row A), lentivirus carrying the GFP gene (row B), or lentivirus carrying the PrPQ167R gene (row C). Frontal tissue sections selected in the panel correspond to the numbers indicated in the schema on the top left. Coloured areas in the schema correspond to anatomical sites referenced for the establishment of vacuolization profiles. Pathological analysis was performed on three independent brains from each category of mice. Bar, 21 µm.

**Figure 5:**
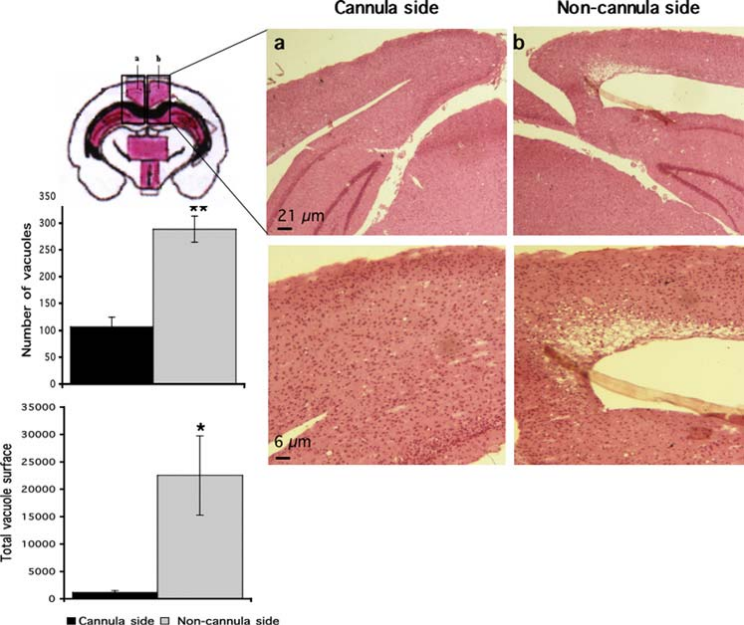
Less spongiosis in the vicinity of the guide cannula in prion-infected mice treated with ΨPrPQ167R. Tissue sections were stained with hematoxylin and eosin. Letters a and b on the schema correspond to the secondary visual cortex (SVC) and a part of the hippocampal CA1 region. Bars, 6 µm. Histograms represent either the mean number of vacuoles or the mean total vacuolar surface in the SVC areas. The number of vacuoles was counted in one square of 250,000 µm^2^, corresponding to nearly all the SVC surface, and repeated in three independent sections. The total vacuolar area was determined by drawing the contour of each vacuole present in one square of 2,500 µm^2^ using Mercator software (Explora Nova, La Rochelle, France), which was repeated in three independent sections from the same mouse. The histograms represent the means±s.e.m. Statistical analysis was performed using STATVIEW 5 software. Values were analyzed by ANOVA followed by the PLSD Fisher test (n = 3). The number of vacuoles in the treated side was statistically different from the non-treated area (**, p<0.005). The total vacuolar area in the cannula side was statistically different from that in the non-cannula side (*, p<0.05).

Tissue sections close to the injection site (coordinates : L: 1.2 mm; R: −1.8 mm; P: −2.0 mm) showed equivalent vacuolisation in the hippocampus and thalamus regions of prion-infected mice treated either with PBS, ΨGFP, or ΨPrPQ167R (Figure 4A–C). However, the mice injected with the dominant negative virions survived 30 days longer than control animals and might have a propensity to develop a higher number of vacuoles and/or larger vacuoles in their brain. A striking difference in spongiosis was observed in the secondary visual cortex (Figure 5, Scheme a–b), an area used to establish vacuolization profiles in standard procedures and located above the injection site (coordinates : L: 0.2 mm; R: −1.8 mm; P: −0.3 mm). A comparison of the secondary visual cortex of both hemispheres showed that there were three times more vacuoles in the non-cannula side of the brain 288±24 than in the cannula side 107±16 (ANOVA, PLSD Fisher test, n = 3, p<0.005). In addition, the vacuoles in the non-cannula side were larger: the total vacuolar area in the treated side was 1,162±333 µm^2^ compared to 22,521±7, 242 µm^2^ in the other side of the brain (ANOVA, PLSD Fisher test, n = 3, p<0.05). The absence of neurodegeneration in the secondary visual cortex suggested that the ΨPrPQ167R had a protective effect on the tissue surrounding the injection site.

### Substantial decrease in spongiosis in the injected side of the brain far from the cannula implantation

**Figure 6:**
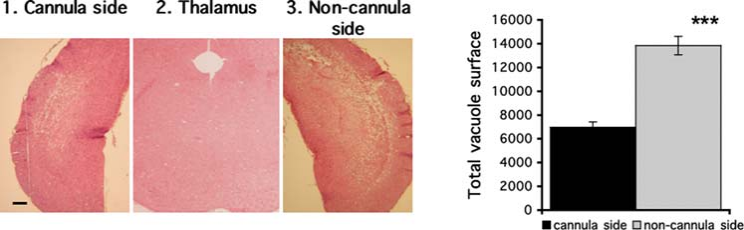
Neurodegeneration in brain regions distant from the guide cannula in prion-infected mice treated with ΨPrPQ167R. Tissue sections were stained with hematoxylin and eosin. (1) and (3) correspond to the ipsilateral and contralateral sides of the brain, respectively, in a coronal section located at 3 mm from the cannula implantation site. (2) corresponds to the thalamus area. Bars, 21 µm. The histogram represents the mean total surface of vacuoles in areas 1 and 3. The total vacuolar area was determined in one mouse by drawing the contour of each vacuole present in four squares of 40,000 µm^2^ each, using Mercator software. The histogram represents the means±s.e.m. Statistical analysis was performed using STATVIEW 5 software. Values were analyzed by ANOVA followed by the PLSD Fisher test (n = 4). The total vacuolar area in the treated side was statistically different from that in the non-treated area (***, p<0.0005).

Tissue sections more distant from the cannula implantation site (R: −4.8 mm, 3 mm distant from the injection site) showed extensive spongiosis that spread into the cortical regions of the brain (Figure 6), probably due to the prolonged survival of the animals. A strong difference was observed between the two hemispheres. We estimated the total vacuolar area on the treated side to be 6,975±436 µm^2^ compared to 13,840±766 µm^2^ on the other side of the brain (ANOVA, PLSD Fisher test, n = 4, p<0.0005) (Figure 6). The enlargement of the vacuoles could be due to the fusion of smaller vacuoles since the animals survived longer.

### Strong decrease in astrocytic gliosis in the brain of mice receiving dominant negative lentiviral vectors

**Figure 7:**
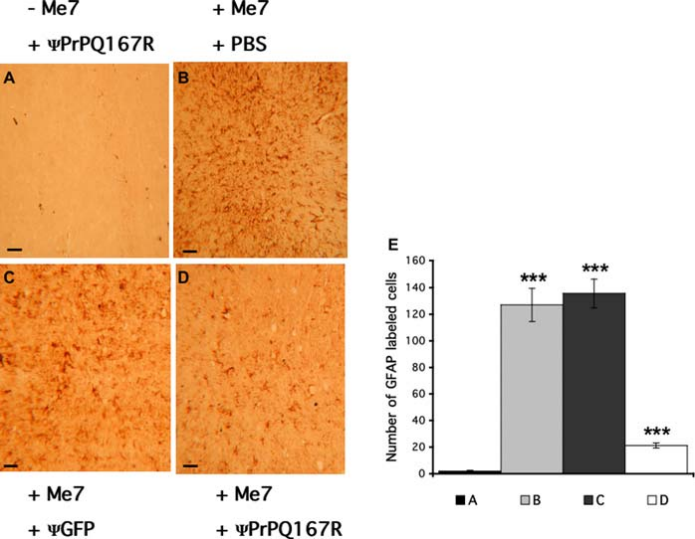
Immunohistochemistry for GFAP in thalamus sections close to the injection site. The plus (+) and minus (−) signs indicate that the mice were or were not inoculated (A), respectively, with the ME7 prion strain. Mice received two injections of either PBS (B) or lentivirus carrying the GFP (C) or PrPQ167R genes (D). GFAP labelling was performed on nine independent brains (3 per group). The sections presented here are representative of the global labelling in the thalamus. Bars, 6 µm. Histograms represent the mean number of GFAP-labelled cells in one mouse of each treated group, which was measured by counting the GFAP-positive cells in five squares of 10,000 µm^2^ each, using Mercator software. The histograms represent the means±s.e.m. Statistical analysis was performed using STATVIEW 5 software. Values were analyzed by ANOVA followed by the PLSD Fisher test (n = 5). Multiple comparison analysis between A, B, C, and D showed that they were all statistically significant (***, p<0.0001), except for A versus D and B versus C.

Immunohistochemistry for GFAP, a marker for inflammation through astrocytic gliosis, was performed in different structures of the mouse brain. Thalamus, as well as hippocampal (data not shown), sections of healthy mice treated with ΨPrPQ167R showed very minor GFAP labelling, illustrating the absence of inflammatory processes that could have been activated by the lentiviral particles (Figure 7A). Me7-inoculated mice receiving either PBS or ΨGFP injections showed high levels of GFAP labelling in thalamus, testifying to the presence of astrocytic gliosis consecutive to the prion disease alone (Figure 7B–C). Surprisingly, Me7-inoculated mice treated with ΨPrPQ167R exhibited a very low rate of GFAP staining (Figure 7D). Estimating the number of GFAP-positive cells with the Mercator software confirmed the histological results. For instance, only 1.8±0.5 GFAP positive cells/10,000 µm^2^ were counted in healthy mice treated with ΨPrPQ167R, while 127±11 and 136±10 GFAP-positive cells/10,000 µm^2^ were counted in prion-infected mice injected with PBS or ΨGFP, respectively (ANOVA, PLSD Fisher test, n = 5, p<0.0001) (Cf. Legend and Figure 7). Surprisingly, prion-infected mice injected with ΨPrPQ167R had 21±2 GFAP-positive cells/10,000 µm^2^, a significant difference from the PBS- and ΨGFP-treated mice (ANOVA, PLSD Fisher test, n = 5, p<0.0001) but not from the healthy mice (ANOVA, PLSD Fisher test, n = 5, p = 0.083). This result shows that prion-infected mice administered dominant negative virions have decreased astrogliosis in their brains.

**Figure 8:**
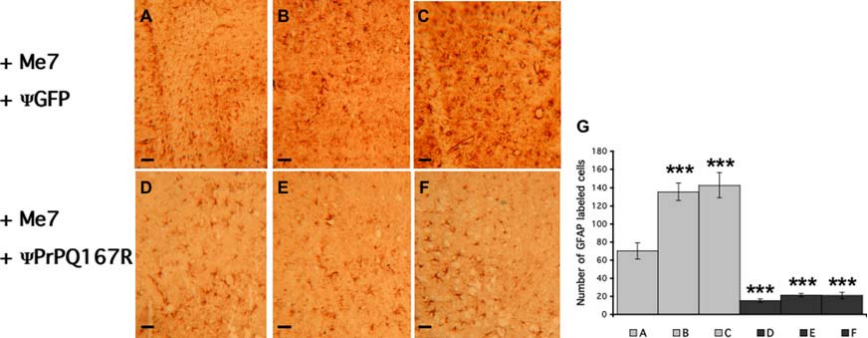
GFAP labelling in tissue sections of prion-infected mice treated with ΨGFP or ΨPrPQ167R. The plus (+) signs indicate that the mice were inoculated with the Me7 prion strain. Mice received two injections of either ΨGFP (A, B and C) or ΨPrPQ167R (D, E and F). The section presented here are representative of the global labelling in the cortex, both in the cannula side (A and D) or non-cannula side (C and F) and in the thalamus (B and E). Bars, 6 µM. The histogram represents the mean number of GFAP-labelled cells in one mouse per group, which was measured by counting GFAP-positive cells in five squares of 10,000 µm^2^ each using Mercator software. The histogram represents the means±s.e.m. Statistical analysis was done using STATVIEW 5 software. Values were analyzed by ANOVA followed by the PLSD Fisher test (n = 5). Multiple comparison analysis among A, B, C, D, E, and F showed that they were all statistically significant (***, p<0.0001), except for B versus C, and D versus E versus F.

Comparative analysis of the GFAP staining in the hippocampus and thalamus was performed in Me7-inoculated mice receiving either ΨGFP or ΨPrPQ167R (Figure 8A–F). The intensity of GFAP labelling in tissue sections from mice treated with ΨGFP increased with increasing distance from the injection site (hippocampus cannula side: 70±9 GFAP cells/10,000 µm^2^; hippocampus non-cannula side: 143±14 GFAP cells/10,000 µm^2^; ANOVA, PLSD Fisher test, n = 5, p<0.0001)(Figure 8A vs 8C; Figure 8G). By contrast, tissue sections from mice treated with ΨPrPQ167R exhibited mild GFAP staining regardless of the location of the injection site (hippocampus cannula side: 15±2 GFAP cells/10,000 µm^2^; hippocampus non-cannula side: 21±4 GFAP cells/10,000 µm^2^, ANOVA, PLSD Fisher test, n = 5, p = 0.6) (Figure 8D vs 8F; Figure 8G). This result is extremely interesting as it suggests either that the lentiviral particles diffused throughout the brain or that the mutant proteins expressed following virion integration, were propagated from cell to cell, possibly through exosomes [37].

## Discussion

Here, we described an innovative gene therapy approach that takes advantage of the natural dominant negative properties of the sheep PrPQ171R and human PrPE219K polymorphisms, both of which are known to be capable of preventing prion diseases. Our aim was also to develop a curative treatment protocol that would be adaptable to human therapeutics, as treatment of CJD patients necessarily starts late. In view of this, we have targeted two critical events during the disease progression: at 35 days p.i. which is the beginning of the neuroinvasion, and at 105 days p.i., when the brain is already deteriorated with presence of spongiosis [34]. Remarkably, ΨPrPQ167R-treated mice at 35 days p.i. survived 19 days (12%) longer than the control group. This result is in good agreement with those of Genoud et al. (2008) [36] who recently reported the use of lentiviral vectors for delivering the soluble prion antagonist PrP-Fc_2_: in their strategy, RML-infected C57Bl/6 mice treated with ΨPrP-Fc_2_ at 30 days p.i., increased their survival time by 25 days (15%). Although the synthetic prion antagonist PrP-Fc_2_ is very different from the natural dominant negative mutant PrPQ167R, those two gene therapy strategies gave very similar results. The small difference in the incubation time is probably due to: (*i*) an infectious dose of prions which is lower than the one we used (3×10^2^ LD_50_ with an incubation time of 177±8 days for PBS-treated mice); (*ii*) a dose of lentivirus much higher (1.5×10^9^ infectious unit compared to 2×10^5^ T.U.), and (*iii*) the treatment that was started earlier during the development of the disease in their model. Genoud et al. (2008) have also observed that a huge administration of ΨPrP-Fc_2_ at 121 days p.i (1.5×10^9^ infectious unit in 30 µl) in mice brain modified the kinetic of disease progression. [36].

Strikingly, ΨPrPQ167R-treated mice at 105 days p.i. survived 9.3 days (6%) more than the control group. Only a single injection was necessary to increase the lifespan of animals and highlights the very potent antagonist effect of the dominant negative ΨPrPQ167R. Such interesting results, along with previous studies showing that the anti-prion effect of dominant negative mutants is dose-dependant in transgenic mice [27], encouraged us to pursue this strategy to enhance protection via chronic injections. Thus, we implemented a system of guide cannula implantation directly into the brain as an alternative to the osmotic pump since lentiviral particles are not stable at room temperature. After just two injections of dominant negative ΨPrPQ167R into prion-infected mice at 80 and 95 days p.i., we observed a prolongation of the incubation time of 30 days, accompanied by a striking improvement in the behaviour of the animals. Importantly, this delay was correlated with: (*i*) a reduction in the spongiosis in the cannula side of the brain which is all the more evident that we are far from the injection site; and (*ii*) a remarkable decrease in astrocytic gliosis throughout the brain. Our results strongly suggest that the ΨPrPQ167R reduced inflammation process in the brain. Together, these results demonstrate that the combination of dominant negative lentiviral vectors and chronic injection into the brain through a guide cannula is a promising approach for the curative treatment of prion diseases.

How does the dominant negative PrPQ167R variant increase the incubation time of the disease? *In vivo* and *in vitro* studies have shown that PrPQ167R cannot itself be converted into its infectious PrP^Sc^ isoform and can also prevent the conversion of wt PrP^C^ into wt PrP^Sc^ via a competitive interaction between PrPQ167R and PrP^C^ for PrP^Sc^ [21], [22], [27]. Dominant negative inhibition in transgenic mice prolongs survival time by slowing the rate of PrP^Sc^ accumulation in infected animals [27]. We have shown here that mice treated with ΨPrPQ167R display a prolonged survival time although the PrP^Sc^ levels in terminally sick mice are not significantly reduced. By contrast to transgenic mice, the presence of substoichiometric PrPQ167R delivered by lentivirus may not be sufficient to completely block PrP^Sc^ formation.

Besides, the substantial decrease of GFAP labelling in the brain suggests that PrPQ167R might play a key role in interfering with the mechanism that activates the inflammatory process. A recent study on prion-infected cells showed that only the PrP^Sc^ isoform interacts with neurons and thereby triggers the recruitment of microglia, leading to upregulated expression of the chemokine RANTES [38]. Activated microglia release various factors, such as cytokines and free radicals, that induce neuronal apoptosis but maintain glial survival [39]. It is possible that ΨPrPQ167R slows down the accumulation of PrP^Sc^ during the disease progression, and as a consequence may diminish the microglia recruitment that activates the inflammatory process.

The prospects for successful gene therapy for prion diseases are often downplayed on the basis that the number of targeted CNS regions is limited, and also because tissue transduction is relatively inefficient and can be constrained by the infectious titer of the virions or the need to target specific cell types. Importantly, our results address these criticisms, as we observed a widespread reduction in astrocytic gliosis, suggesting that the lentiviral particles and/or the dominant negative proteins were able to diffuse far from the injection site which was also observed by Genoud et *al.* (2008) in their study [36]. Protein propagation would likely occur through cell to cell contacts involving small cellular vesicles of endosomal origin called exosomes, which have been proposed to contribute to the spread of prions [37]. A mechanism proposed by Genoud et *al.*, to explain the neuroprotective effect far from the injection site is the propagation of anti-prion antagonist proteins *via* a noncell autonomous manner [36].

Importantly, this gene therapy approach is effective even when started at a late stage of prion infection. Our results thus represent a promising advance towards a possible curative treatment for prion diseases in humans.

## Supporting Information

Movie S1 Healthy C57Bl/6 mouse with a guide cannula implanted into its brain and treated with PrPQ167R virions. Movie was recorded at 172 days post-infection with prions, on a mouse representative from the group.
(0.91 MB MOV)

Movie S2 Prion-inoculated C57Bl/6 mouse with a guide cannula implanted into its brain and treated with GFP virions. This animal is representative from a group of mice that was treated with GFP virions at 80 and 95 days post-infection (p.i.) with prions. The movie was recorded at 172 days p.i. corresponding to the terminal stage of the disease.
(0.91 MB MOV)

Movie S3 Prion-inoculated C57Bl/6 mouse with a guide cannula implanted into its brain and treated with dominant negative PrPQ167R virions. This animal is representative from a group of mice that was treated with PrPQ167R virions at 80 and 95 days post-infection (p.i.) with prions. The movie was recorded at 172 days p.i. that is the moment when control groups have been killed.
(1.21 MB MOV)

Movie S4 Prion-inoculated C57Bl/6 mouse with a guide cannula implanted into its brain and treated with dominant negative PrPQ167R virions. This animal is representative from a group of mice that was treated with PrPQ167R virions at 80 and 95 days post-infection (p.i.) with prions, and presenting survival times lasting from 182 days p.i. to 193 days p.i. The movie was recorded at 189 days p.i. at the terminal stage of the disease.
(0.61 MB MOV)
